# Nuclear Markers of Danube Sturgeons Hybridization

**DOI:** 10.3390/ijms12106796

**Published:** 2011-10-14

**Authors:** Andreea Dudu, Radu Suciu, Marian Paraschiv, Sergiu Emil Georgescu, Marieta Costache, Patrick Berrebi

**Affiliations:** 1 Department of Biochemistry and Molecular Biology, University of Bucharest, Splaiul Independentei, Bucharest 91-95, 050095, Romania; E-Mails: tn_andreea@yahoo.com (A.D.); georgescu_se@yahoo.com (S.E.G.); 2 Sturgeon Research Group, Danube Delta National Institute, 165 Babadag Street, Tulcea 820112, Romania; E-Mails: radu@indd.tim.ro (R.S.); mparaschiv@indd.tim.ro (M.P.); 3 Institut des Sciences de l’Evolution, (UMR UM2-CNRS-IRD 5554) University Montpellier II, cc 065. Place E. Bataillon, 34095 Montpellier Cedex 5, France; E-Mail: patrick.berrebi@univ-montp2.fr

**Keywords:** sturgeons, microsatellites, hybrids, statistical analysis

## Abstract

Acipenseriformes are composed of 25 sturgeon species and two paddlefish species distributed exclusively in the northern hemisphere. The Danube River and the Black Sea were originally inhabited by six sturgeon species but two are extinct and only four are still reproducing currently in the Lower Danube: *Huso huso*, *Acipenser stellatus*, *A. gueldenstaedtii* and *A. ruthenus*. Sturgeon species hybridize more easily than other fish and the determination of pure species or hybrid status is important for conservation and for breeding in fish farms. This survey demonstrated that morphological determination of this status is not reliable and a molecular tool, based on eight microsatellites genotypes is proposed. This method, based on three successive statistical analyses including Factorial Correspondence Analysis (FCA), Structure assignation and NewHybrids status determination, showed a high efficiency in discriminating pure species specimens from F1, F2 and two kinds of backcross individuals involving three of the four reproducing Lower Danube sturgeon species.

## 1. Introduction

Sturgeons represent one of the oldest groups of fish in the world originating in the Jurassic period [1] and which has successfully survived several mass extinction events during their history. Due to their great economical value, sturgeons have been fully exploited by overharvesting and poaching. It is considered that some factors in their life history such as their long lifespan, delayed maturation or anadromy, which has made them resilient to global environmental modifications in the past, are now responsible for their high susceptibility to extinction under anthropogenic impact [2].

Acipenseriformes are distributed exclusively in the northern hemisphere, with a higher concentration of species in the Ponto-Caspian region [1]. The Danube River and the Black Sea were originally inhabited by six sturgeon species [3]: the beluga sturgeon (*Huso huso*), the Russian sturgeon (*Acipenser gueldenstaedtii*), the stellate sturgeon (*A. stellatus*), the ship sturgeon (*A. nudiventris*), the freshwater sterlet (*A. ruthenus*) and the European sturgeon (*A. sturio*).

Except for *A. sturio* which is a basal species in the phylogeny of the genus, the Danubian sturgeons are representatives of the Atlantic clade, including *H. huso* whose genus should be changed. According to the Peng et al. [4] phylogeny based on cytochrome b sequences, the Atlantic clade is structured into one basal species (*H. huso*) and then two clades, a diploid one (composed of 3 species: *A. stellatus*, *A. nudiventris* and *A. ruthenus*) and a tetraploid one (composed of 6 species, among them *Acipenser gueldenstaedtii*).

In the past, sturgeons from the Black Sea, especially the beluga sturgeon, used to migrate upstream in the Danube River up to Budapest, Vienna, and even in Bavaria. Nowadays the migration is interrupted at the Iron Gate Dams (Romania), which has severely affected the sturgeon populations from the Middle and Upper Danube [5,6]. The Lower Danube River (downstream of the Iron Gate dams) is now considered to be the last refuge for sturgeons in the Black Sea basin. From the six sturgeon species mentioned above, only four are still reproducing currently in the Lower Danube: *H. huso*, *A. stellatus*, *A. gueldenstaedtii* and *A. ruthenus*. Both European and ship sturgeon are considered extinct populations in this region [3].

Nowadays, exotic sturgeons are used in local fish farms, producing artificial hybrids. Such hybrids can be found in the Danube [7].

All *Acipenseriformes* are divided into three separate groups depending on the number of chromosomes: (1) species with karyotypes comprising about 120 chromosomes; (2) species with 240 to 270 chromosomes; they are conventionally referred to as 250-chromosomes species; (3) species with around 370 chromosomes [8]. Two scales of Acipenseriformes ploidy have been proposed: (1) the “evolutionary scale”: diploid species (extinct), tetraploid species (120-chromosomes), octoploid (250 chromosomes), and 12-ploid (370-chromosomes) species [9]; and (2) the “contemporary scale”: diploid (120-chromosomes), tetraploid (250-chromosomes), and hexaploid (370-chromosomes) species [10].

Sturgeons are known to interbreed under natural conditions, giving rise to viable and sometimes fertile interspecific and intergeneric hybrids. The main reason for hybridization is the overlap in time and space of breeding areas which is increased by the reduction of the reproductive sites in Danube River due the anthropic intervention. For a long period of time, sturgeon hybrids were considered separate species or subspecies. For instance, *A. nasus* Heckel, 1851, is in fact the hybrid between *H. huso* and *A. naccarii* and *A. primigenius* Chalikov, 1941, represents the hybrid of *A. ruthenus* and *A. gueldenstaedtii.*

It was observed that artificially obtained sturgeon female hybrids of the first generation between the parental species of the same ploidy can produce eggs, while hybrids from the crosses of parental species of different ploidy have reduced fertility or are sterile [11].

Until now, hybrids identification was done using only morphological characters taking into consideration that the hybrids inherit morphological features from the parental species. Still, the identification of a certain individual as a hybrid, based only on morphology, is not reliable enough and becomes really difficult when three species are involved, as in the case of crosses between a bester (*H. huso* × *A. ruthenus)* and *A. gueldenstaedtii*. Furthermore, the identification of juveniles based only on morphometric data is proved to be more difficult than adult hybrids identification. Only a genetic study can provide the necessary proof that nuclear genes from both parental species are present in the hybrid.

Due to their characteristics (high polymorphism, high power of discrimination, codominant mendelian inheritance, *etc*.), nuclear markers as microsatellites have been proven to be very useful for identifying sturgeon hybrids taking into account that the alleles of an individual represent a combination of parental alleles. Several methods were proposed for identifying hybrid individuals [12–14]. One of these methods for pure species and hybrid identification is based on the use of alleles that are present in one species only (diagnostic alleles) or of microsatellite loci that are fixed for alternate alleles in different species (diagnostic loci). Other statistical methods using microsatellite data do not necessarily require that the different species possess unique alleles because they are based on the probability of individual genotypes at multiple loci to belong to parental species, taking into account the parental species allele frequencies [12].

Until now there are only a few studies in the literature investigating the hybridization in sturgeons based on microsatellite markers [7,14–16]. Danube sturgeon molecular studies for hybrids characterization are still missing. Establishing a reliable molecular method for hybrids identification is very important considering the conservation programs that aim at the restoration of Danube sturgeon populations. The introduction, through these programs, of hybrid genitors and releasing of hybrid juveniles incorrectly labeled as pure individuals might have an unfortunate impact on the genetic diversity of sturgeon stocks.

This study represents the first attempt in finding a reliable tool based on nuclear markers for Danube sturgeon hybrids and it aims at: (i) the selection of suitable microsatellites loci among the numerous published options; (ii) the calculation of allelic frequencies of the selected loci in Danube natural populations through referencing pure individuals of the four species; (iii) the design of a statistical method able to estimate the nature and level of hybridization of any sturgeon in the Lower Danube River. For this, 84 sturgeons were analyzed and 25 expected microsatellites loci checked.

## 2. Results and Discussion

### 2.1. Microsatellite Loci Selection

Among a total of 25 microsatellite primers pairs tested, we obtained reproducible amplification in all four species of Danube sturgeons for ten loci. Among these ten amplifiable loci, eight showed interspecific polymorphism (LS19, LS34, LS39, LS54, Aox27, AoxD234, AnacE4 and AnacC11) and were conserved for analyses, while two were totally monomorphic in the four species (As002 and AciG98). The other fifteen loci displayed difficulty in amplification, in which case the correct reading of the allelic profile is very difficult (Supplementary Table ST2).

The eight microsatellites selected for further analysis displayed good amplification in all four investigated sturgeon species, reproducibility of amplicons within individuals and interspecific polymorphism.

### 2.2. Allelic Frequencies

The total number of alleles observed at each locus ranged from 1 to 8 (*A. stellatus* and *A. gueldenstaedtii*), 2–11 (*H. huso*) and 1–5 (*A. ruthenus*) (Table 1). The allelic frequencies were calculated and potential private alleles with value of diagnostic alleles were recorded. The diagnostic alleles are distributed in a range of frequencies, from 0.01 to 1.

### 2.3. Multidimensional Analysis (FCA)

In a first step, the genotypes data were run in a FCA test, using Genetix software, only for the individuals considered to be pure species. The FCA highlighted the differences between the four analyzed species. Four main clusters, each corresponding to one of the sturgeon species analyzed were identified. The most informative is axis 1 (10.41% of the total genetic variation) which separates the four species but especially *A. stellatus* against the three other ones. The second axis, a little less informative (9.81%), separates *H. huso* and *A. ruthenus*, followed by axis 3 separating mainly *H. huso* and *A. gueldenstaedtii* with 7.11% of total genetic variation (Supplementary Figure SF1).

Some of the individuals considered as pure species according to their morphology (Agu259, Agu248, Hh247, Ast0599) fall out of their cluster. This might be considered as a first indication that these individuals were incorrectly labeled as pure species and, in fact, they might be hybrids to be tested (Supplementary Figure SF1).

In a second step, the genotypes data of putative hybrids were included in FCA as supplementary individuals. The analysis highlighted a fifth category including the putative hybrids, but also individuals that were considered as pure species, based on their morphology (Supplementary Figure SF2). Taking into consideration that, according to the multidimensional analysis (confirmed by the next method, see Section 2.4.), only three of the four analyzed species hybridize each other, the putative hybrids displaying an intermediate position between *A. stellatus*, *H. huso* and *A. gueldenstaedtii*. Consequently, we deduced that *A. ruthenus* individuals should be eliminated from further FCA tests.

**Figure 1:**
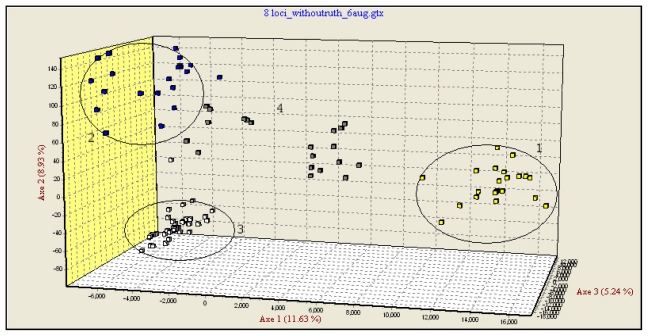
Factorial Correspondence Analysis (FCA) based on eight microsatellite loci in three pure species and hybrids of Danube sturgeons: (1) *A. stellatus*; (2) *H. huso*; (3) *A. gueldenstaedtii*; (4) Hybrids and some pure individuals.

In the third step, multidimensional analysis was performed only on three pure species and hybrid individuals as supplementary elements (Figure 1). The new FCA analysis grouped the pure species and the hybrids in distinct clusters. The hybrids and some of the individuals labeled as pure were occupying an intermediate position between the pure species groups, some of these being placed approximately in the middle of the triangle delimited by the three pure species, while others appear to be closer to *H. huso* and *A. gueldenstaedtii*.

### 2.4. Assignment Test with Structure

**Figure 2:**
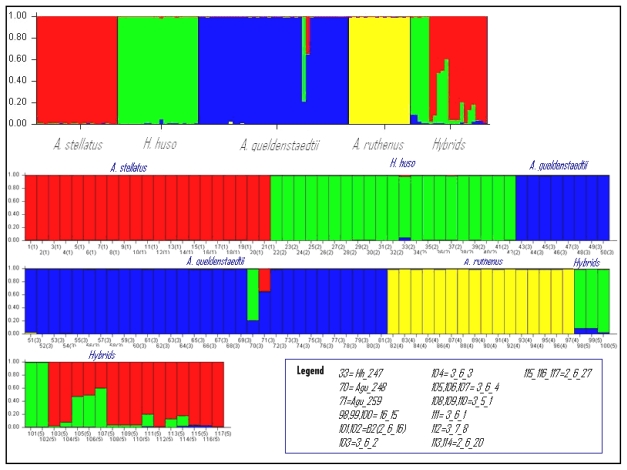
Assignation of the 84 sturgeons by Structure analysis based on eight microsatellite loci in four sturgeon species from Danube. Histograms represent the estimated membership coefficients (Q). Composite bars are expected hybrids.

The assignment test conducted with Structure for K from 2 to 6 confirmed the presence of five specific clusters: (1) *A. stellatus* pure species; (2) *H. huso* pure species; (3) *A. gueldenstaedtii* pure species; (4) *A. ruthenus* pure species; (5) hybrids (Figure 2). The pure individuals (with three exceptions) were strongly assigned in their corresponding species. The K value was determined when the four morphological species were clearly clustered (Figure 2). The perfect correspondence between morphological and molecular/assignation determinations gives a great reliability to the results. The Q values for the pure individuals in the ten independent runs were very similar and the mean Q value for the pure species cluster was greater than 0.98. Individuals Hh_247 (Q = 0.94), Agu_248 (Q = 0.211) and Agu_259 (Q = 0.643) showed lower values of membership coefficient than 0.95 and this might lead to the hypothesis that these individuals are hybrids and in this case the assignment by Structure is in accordance with the multidimensional analysis (Supplementary Table ST3).

### 2.5. Determination of Hybrid Categories

The hybrids confirmed by the FCA and Structure assignment test were analyzed together with their two genitor species using the NewHybrids software in order to distinguish between F1 and later hybridization steps.

The first test included *A. stellatus*, *H. huso* and their hybrids. The posterior probabilities for each individual to belong to one of the six categories (pure *A. stellatus*, pure *H. huso*, F1, F2, backcross1 and backcross2) are shown in the Supplementary Table ST4. All individuals that were labeled as *A. stellatus* and *H. huso* showed a posterior probability higher than 0.97 to be composed of one of the two species. Regarding the hybrids between these two species, 37.5% are F1 hybrids and the remaining 62.5% belong to *A. stellatus* back cross category.

The second test included *A. gueldenstaedtii*, *H. huso* and their hybrids. The posterior probability for *H. huso* individuals to be pure was higher than 0.97, with the exception of three individuals: Hh 247 (0.887), Hh_278 (0.885) and Hh_276 (0.880). In the case of *A. gueldenstaedtii*, all individuals presented a probability >0.98 to be pure, except the Agu_248 with a 0.634 probability to be in backcross 2 category (Supplementary Table ST5).

The third test included *A. stellatus*, *A. gueldenstaedtii* and their hybrids. The posterior probability that individuals included in this analysis are pure was higher than 0.97 with the exception of Agu_259, which showed a probability of 0.990 to be a F2 hybrid (Supplementary Table ST6).

Such methods have been frequently used for hybrid detection and introgression measurements, but the three methods have never been performed together. Among very recent analyses, some authors, in a context of marine hybrid zone, used both FCA and Structure programs [17]. Lajbner *et al*. [18] mainly used NewHybrids.

### 2.6. Hybrids Categories Based on Morphology and Statistical Analyses

Based on morphological observations, several hybrid sturgeons were identified in the Danube River, between 2001 and 2008. For this, ventral photos of heads (Supplementary Figures SF3 and SF5) of suspected young of the year hybrids and potential parental species were taken in the field or in the laboratory with the aim of linking morphology of the heads to genetics analysis. In some cases karyological analyses were conducted (Supplementary Figure SF4) using either *in vivo* “air dried” chromosome preparation method from kidney tissue [19] or *in vitro* lymphocyte cultures from sterile blood samples [20]. All these current observations lead to what is called here morphological determination mostly of pure species but sometimes of hybrids.

The use of diagnostic alleles’ presence as a basis of hybridization detection was tested here but this test was not considered efficient. Although diagnostic alleles from both genitor species were identified in all hybrids, the presence of this type of alleles cannot be considered as a reliable tool, taking into consideration the limited number of pure species reference individuals analyzed (16 to 22). There is the risk that an allele considered as private for a species might occur in other species when the number of analyzed individuals is increased.

The statistical detection of hybrids is secure because of the nearly perfect correspondence (see Figure 1 and 2) between the two methods based on totally different concepts. Then, the treatments of the data by three successive steps were, in the majority of the cases, in agreement with their morphological classification. Some exceptions, described below, plainly justify the use of the molecular tool.

#### 1) Morphological pure species/molecular hybrids

There were two cases (Agu_248 and Agu_259), considered as pure *A. gueldenstaedtii* individuals according to morphology, that were not confirmed by molecular data.

According to multidimensional analysis the individual **Agu_248** is placed together with individuals morphologically labeled as hybrids and it occupied an intermediate position between *H. huso* and *A. stellatus*. The Structure analysis showed that this individual should be composed of 0.211 of *A. gueldenstaedtii* and 0.783 of *H. huso.* The posterior probability value obtained in NewHybrids is 0.634 probably for a backcross2, which means that its parents’ origin should be a F1 hybrid *A. gueldenstaedtii*/*H. huso* backcrossed with *H. huso*, this fact being confirmed by the Structure probabilities situated around 25% *A. gueldenstaedtii*/75% *H. huso*. The classification of this individual as a backcross2 hybrid is in opposition with literature data suggesting that hybrids resulted from the crosses of parental species with different ploidy (which is the case of A. *gueldenstaedtii* and *H. huso*) showed decreased fertility or complete sterility [8,11,21]. The individual **Agu_259**, according to FCA and Structure test, should be a hybrid between *A. gueldenstaedtii* and *A. stellatus*, the genotype composition values in this case being 0.64 for *A. gueldenstaedtii* and 0.34 for *A. stellatus.* The NewHybrids test placed this individual in the F2 category with a posterior probability of 0.990.

These are the first cases indicating that sturgeon individuals considered as pure *A. gueldenstaedtii*, based only on morphological features, can be misleading and that molecular data provide a correct diagnostic.

#### 2) Morphological hybrids/molecular pure species

In contrast, two individuals initially considered as undetermined hybrids, were classified as pure species according to statistical analyses applied on molecular data. Thus, individuals **B2 (2_6_16)** and **2_6_27** appear to be respectively pure *H. huso*, and pure *A. stellatus*, proving that atypical morphological characteristics of a pure sturgeon can lead to an incorrect hybrid diagnostic.

#### 3) Pure species individuals misclassified by FCA

The individual **Hh_247** that was labeled as pure *H. huso*, based on morphology, was placed by the FCA analysis in the hybrids category. Although the Q value (0.94) and the posterior probability (0.878) were lower than for pure *H. huso* individuals, both Structure and NewHybrids analyses considered this individual as pure.

The individuals **7_11_12**, **7_11_15** and **Hh_280** were confirmed by both Bayesian statistical methods implemented in Structure and NewHybrids as pure individuals despite the fact that the FCA placed them outside the cluster of *H. huso*. The same situation was observed for individual **Ast_0599** which was confirmed as a pure *A. stellatus* with a probability of 0.99 by Structure. These observations suggest that the specific delimitation overlaps in FCA analysis. Therefore these cases demonstrated that an individual cannot be classified as pure or hybrid based only on one method, cumulating the results of several methods only confers an acute final diagnostic.

#### 4) High precision hybrid status

The hybrid **3_6_4** is confirmed by all three statistical tests as a hybrid between *A. stellatus* and *H. huso.* Structure genotype composition is expected to be 0.472 and 0.523 respectively, which represents an F1 hybrid, determination confirmed by a posterior probability of 0.958 for this status obtained in NewHybrids test.

Individual **16/15** (Supplementary Figure SF3**)**, captured in the Black Sea, represents a hybrid between *H. huso* and *A. gueldenstaedtii*, a fact confirmed by all statistical results. The NewHybrids analysis placed this individual in the backcross2 category, which means that it is a product of F1 hybrid between *H. huso* and *A. gueldenstaedtii* which backcrossed with *H. huso*. As a confirmation, chromosome counts in individual 16/15 (Supplementary Figure SF4) revealed that it had an intermediary number 2n = 180 ± 4, between *H. huso* (2n = 120) and *A. gueldenstaedtii* (2n = 240), in accordance with findings in artificially produced hybrids [22].

#### 5) Low precision: higher number of microsatellite loci is needed

The analyses pointed out some inaccuracies, mainly between multidimensional analysis and NewHybrids results and Structure results, but not solely. Individuals **2_6_10**, **3_6_3**, **3_6_11** and **3_7_8** were classified as hybrids between *A. stellatus* and *H. huso*, by FCA and NewHybrids test. According to the latter, they belong to backcross2 category, as results of backcrossing between an F1 *A. stellatus*/*H. huso* and a pure *A. stellatus*. This classification was confirmed by Structure results for 2_6_10 and 3_6_11 individuals. However, 3_6_3 and 3_7_8 were assigned to pure *A. stellatus* with a genotype composition >90%.

According to FCA and NewHybrids tests individuals **3_6_2** and **3_5_1** might be considered as F1 hybrids between *A. stellatus* and *H. huso*, but this is not sustained by the Q values obtained in Structure giving an *A. stellatus* composition of 0.980 and 0.965 respectively.

The differences obtained between the statistical methods used in this study are limited to individual hybrid status, but are never concerned with whether a given individual is a hybrid or not, nor with the species involved in the hybridization. It might be an indication that a higher number of microsatellite loci should be analyzed, with a higher number of pure individuals as species references, in order to have a better estimation of the real genetic diversity of each species.

Based on our results, the use of molecular data to assess the genetic structure in Danube sturgeon individuals is necessary because a classification as pure species or as hybrids, based only on morphological data, can be misleading and inaccurate. In addition, the analysis of genotypic data using only one statistical method can be risky and can result in inaccurate or wrong interpretations. In fact, the sum of all methods can be reliable and offers the final confirmed diagnostic.

The method proposed here might be considered as a good tool for sturgeon hybrids identification, but could be improved by a higher number of pure species reference individuals and of microsatellite loci.

## 3. Experimental Section

### 3.1. Sample Collection

Biological samples (fin tissue) were collected from 84 sturgeon individuals captured in Romania in the Lower Danube River or in the Black Sea coastal waters, including 22 expected *A. stellatus* (captured from 2001 to 2007), 21 expected *H. huso* (2001), 16 expected *A. gueldenstaedtii* (2007–2008), 16 expected *A. ruthenus* (2007–2008), 7 expected hybrid specimens (2003) and 2 undetermined young specimens (1996). The individuals were morphologically characterized according to CITES identification guide [23].

### 3.2. Selection of Microsatellite Loci

Total genomic DNA was extracted from small pieces of fin tissue using the Chelex extraction method [24]. Twenty-five sets of published microsatellite primers were tested on several expected pure individuals of each Danube sturgeon species. Six loci were developed for shovelnose sturgeon (Spl100, Spl101, Spl104, Spl106, Spl120, Spl163 [25]), five for lake sturgeon (LS19, LS34, LS39, LS54, LS68 [26]), five for gulf sturgeon (Aox23, Aox27, Aox45 [27] and AoxD161, AoxD234 [28]), five for Adriatic sturgeon (An20 [29] and AnacE4, AnacG8, AnacB11, AnacC11 [30]), two for Chinese sturgeon (As002, As004 [31]) and two for green sturgeon (AciG56, AciG198 [32]).

The amplifications were performed in a final volume of 20 μL. Each reaction mixture contained template DNA, 1X GoTaq Reaction Buffer (Promega), 1.5–3 mM MgCl_2_, 0.4 U of GoTaq Polymerase (Promega), 0.4 μM of each primer, 200 μM of each dNTPs. Cycling conditions were as follows (except for loci LS39 and An20): denaturation at 95 °C for 5 min; then 35 cycles of 30 s at 95 °C, 30–45 s at Tm and 1 min at 72 °C; and a final extension at 72 °C for 5 min.

For locus LS39, in order to eliminate the unspecific amplification, the following TouchDown PCR was performed: 95 °C for 5 min; 10 cycles: 30 s at 95 °C, 30 s at 67–57 °C (using a 1 °C decrease in each cycle), 30 s at 72 °C; 25 cycles: 30 s at 95 °C, 30 s at 57 °C, 30 s at 72 °C, and a final extension at 72 °C for 5 min.

For locus An20, a TouchDown PCR was also performed using the following conditions 95 °C for 5 min; 8 cycles: 30 s at 95 °C, 30 s at 58–54.5 °C (using a 0.5 °C decrease in each cycle), 30 s at 72 °C; 27 cycles: 30 s at 95 °C, 30 s at 54 °C, 30 s at 72 °C, and a final extension at 72 °C for 5 min. (Supplementary Table ST1).

The loci that showed a good amplification were analyzed on polyacrylamide gel using as fluorescent general staining 0.1X SYBR Gold (Invitrogen). The gels were scanned using FMBIO II Fluorescence Imaging System (Hitachi) and the loci were visually analyzed without scoring the alleles in a preliminary step.

### 3.3. Microsatellites Genotyping

Finally, a set of eight microsatellite markers (LS19, LS34, LS39, LS54, Aox27, AnacC11, AnacE4 and AoxD234) were selected for genotyping. PCR was performed using fluorescent labeled primers and the amplification conditions described in Supplementary Table ST1. PCR products were separated by electrophoresis on PAA gel and scanned in FMBIO II Fluorescence Imaging System (Hitachi). The allele size scoring was accomplished using the FMBIO II ReadImage software.

Some loci had a tetraploid pattern for *A. gueldenstaedtii* only, which is considered to be a tetraploid species. For tetraploid species, the determination of the exact genotype is difficult and furthermore, there is no statistical method that permits the analysis of tetraploid data together with the diploid ones. Because the aim of this study is the hybrid identification, no determination of the “true” genotypes for polyploid patterns and no quantification were achieved in this way. Thus, polyploid genotypes were transformed into several diploid ones. For example, if an individual had three alleles A, B and C, this individual was multiplied into three artificial diploid individuals, each wearing one genotype AB, BC or AC, in order to analyze the data without missing any information. Of course, using this artifice, the neither true allelic frequencies nor panmixia be properly estimated, but this is the logical consequence of our inability to determine the true tetraploid genotypes.

### 3.4. Statistical Analysis of Microsatellite Data

In order to propose a tool able to detect and characterize any kind of hybrid between the four sturgeon species considered, a four-step method has been tested.

Step 1. Allelic frequencies of pure species samples were calculated with Genetix software [33] and the occurrence of species-specific alleles was recorded.

Step 2. A Factorial Correspondences Analysis (FCA) was done with Genetix in order to investigate the relationships among individuals. This type of analysis can explain a maximal amount of genetic variation using a minimal number of factors and it can provide the means for visualizing the genetic relationships between populations/species.

Step 3. Assignment tests were performed with Structure software version 2.1 [34]. Ten independent runs with 500,000 burnins and 1,000,000 Markov Chain Monte Carlo (MCMC) iterations were performed for *k* = 4 (number of pure species analyzed in this study). The analysis was done assuming correlated allele frequencies and admixture.

Step 4. The hybrids, confirmed by the multidimensional analysis and the assignment test, were analyzed together with their genitor species with the NewHybrids software [35]. This method, apart from the hybrid identification, computes the posterior probability that an individual belongs to a hybrid category (F1, F2 or backcrosses). The analysis was done using 500,000 permutations.

## 4. Conclusions

Decisions to initiate repopulation programs for the purpose of conservation of these valuable species, need to take into account, besides the socio-economical aspects, the assessment of genetic diversity and the correct diagnostic of fishes included in this type of program. Based on our results, the use of molecular data to assess the genetic structure in Danube sturgeon individuals is necessary because a classification as pure species or as hybrids, based only on morphological data, can be misleading and inaccurate. Also, the analysis of genotypic data using only one statistical method can be risky and can result in inaccurate or wrong interpretations. However, the sum of utilizing all methods has proven to be reliable, and offers the final confirmed diagnostic. The method proposed here might be considered a good tool for sturgeon hybrids identification, while it can be improved by including a higher number of loci and of reference individuals.

## Supplementary Material



## Figures and Tables

**Table 1:** Characteristics of eight microsatellite loci selected for hybrids diagnostic.

	Alelle Size Range (bp)				Total Number of Alleles (A)			
Locus	*A. stellatus*	*H. huso*	*A. gueldenstaedtii*	*A. ruthenus*	*A. stellatus*	*H. huso*	*A. gueldenstaedtii*	*A. ruthenus*
LS19	133–145	133–163	133–157	136–142	5	9	7	3
LS34	148	142–148	142	139–145	1	2	1	2
LS39	96	105–114	105–108	102–108	1	4	2	3
LS54	140–192	220–252	196–215	152–160	8	7	6	2
Aox27	126–146	122–134	114–150	118	6	4	5	1
AoxD234	203–247	195–251	207–271	219–271	6	11	8	5
AnacE4	346–358	332–360	326–360	326–332	6	6	7	3
AnacC11	153–193	145–173	165–189	161–201	6	3	4	5
Total number					39	46	40	24
