# Supplementary material for: Nuclear Markers of Danube Sturgeons Hybridization
Source: Int J Mol Sci. 2011 Oct 14;12(10):6796–809. doi: 10.3390/ijms12106796 (PMC3211011; doi:10.3390/ijms12106796)
Supplement: Supplementary file 1 [file ijms-12-06796-s001.doc]

Supplementary material

Figure SF1. Factorial Correspondence Analysis (FCA) based on 8 microsatellite loci in the four pure species of
Danube sturgeons.


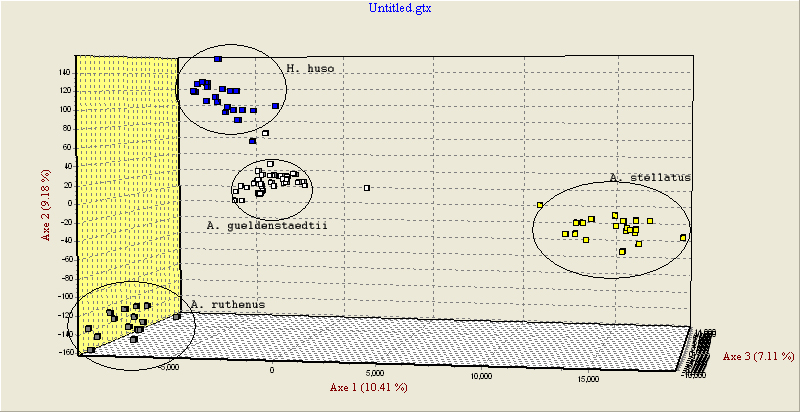


Figure SF2. Factorial Correspondence Analysis (FCA) based on 8 microsatellite loci in the four pure species and hybrids of Danube sturgeons. (1) *A. stellatus*; (2) *H. huso*; (3) *A. gueldenstaedtii*; (4) *A. ruthenus*; (5) Hybrids and some pure individuals to be tested with
other methods.


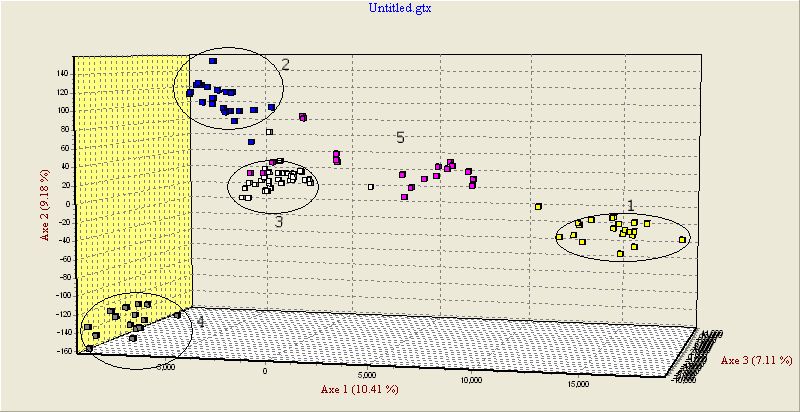


Figure SF 3. Young sturgeons 16/8 (*A. gueldenstaedtii*) and 16/15 (hybrid *A. gueldenstaedtii x H. huso*) captured on 10^th^ of May 1996 at Ciotica, Black Sea.


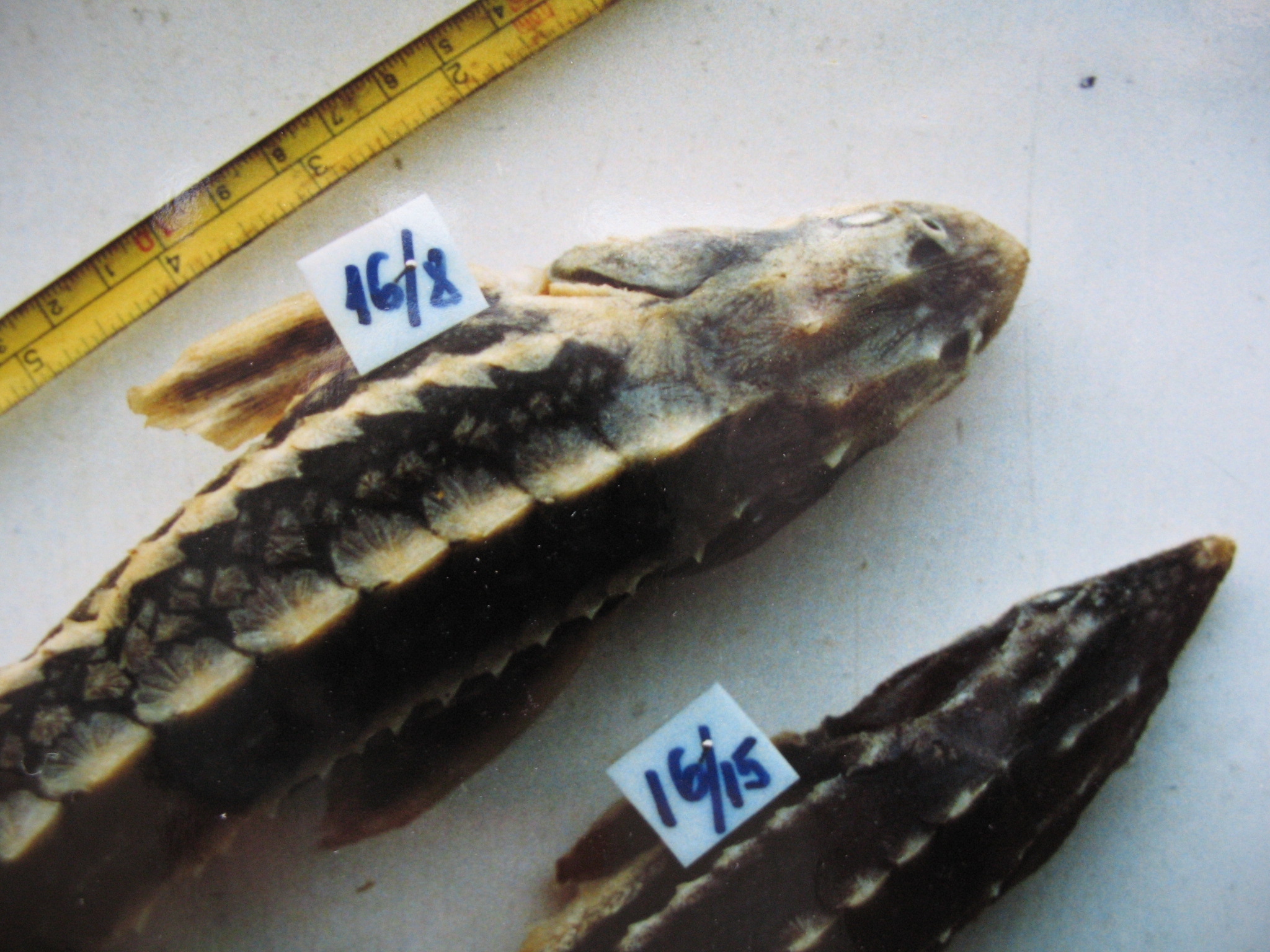

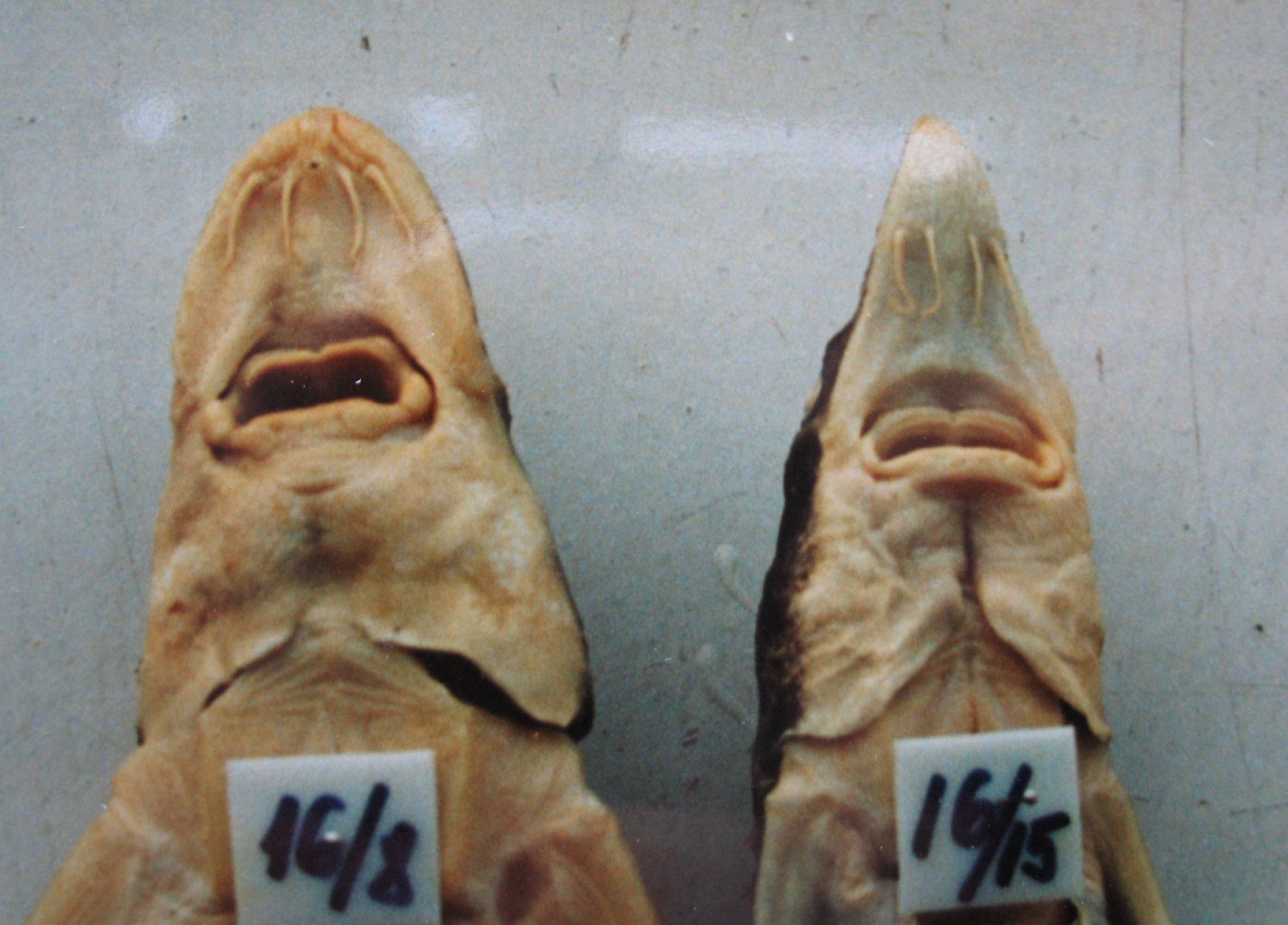


**Figure SF 4.** Metaphase and karyotype of young sturgeon 16/15 captured on 10^th^ of May 1996 at Ciotica, Black Sea (2n = 180).


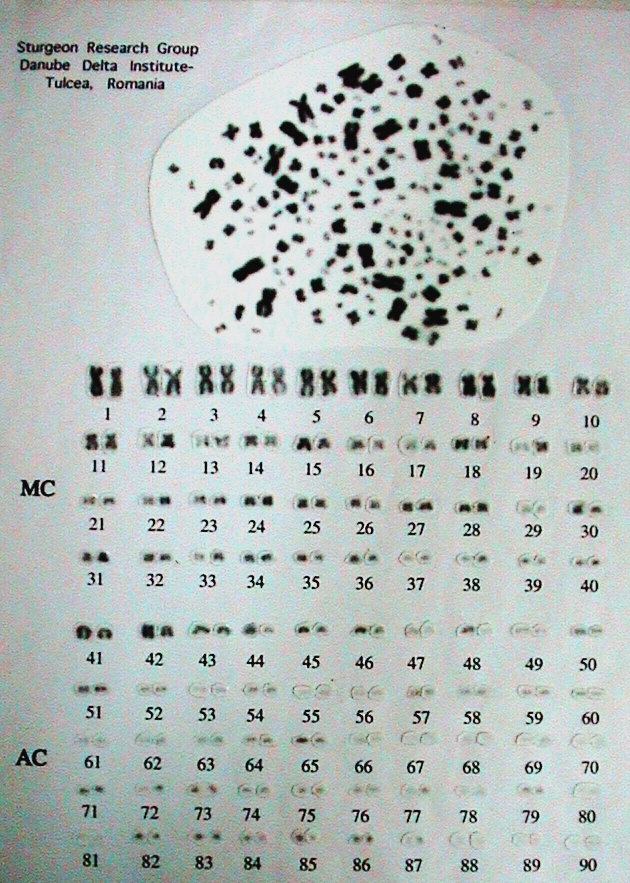


Figure SF 5. Young sturgeons captured in June 2003 at Danube River Km 123:
left: *A. stellatus*; middle: hybrid *A. stellatus x H. huso*; right: *H. huso.*


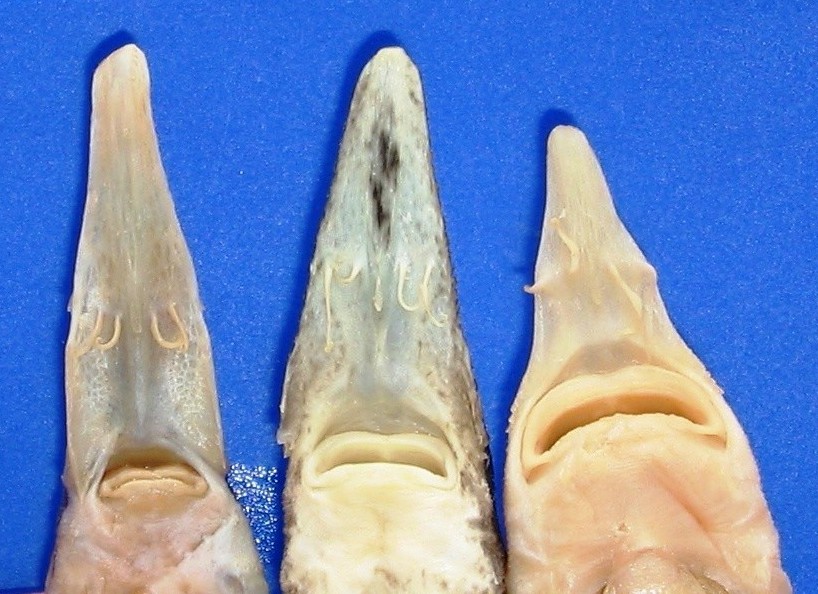


Table ST1. Microsatellite loci analyzed in this study.

| **Locus** | **Species** | **Reference** | **Repeat motif** | **GenBank**  **accession no.** | **Fragment size** | **Primer sequence from 5' to 3'** | **Annealing**  **temperature**  **(ºC)** | **Annealing time (sec)** |
| --- | --- | --- | --- | --- | --- | --- | --- | --- |
| LS19 | *A. fulvescens* | May et al., 1997 | (TTG)9 | U72730 | 118-145 | F: *CATCTTAGCCGTCTGTGGTAC  R: CAGGTCCCTAATACAATGGC | 55 | 30 |
| LS34 | *A. fulvescens* | May et al., 1997 | (GTT)10 | U72733 | 114-156 | F*: TACATACCTTCTGCAACG  R:GATCCCTTCTGTTATCAAC | 55 | 30 |
| LS 54 | *A. fulvescens* | May et al., 1997 | (GATA)6  (GACA)7 | U72735 | 180-192 | F:*CTCTAGTCTTTGTTGATTACAG  R: CAAAGGACTTGAAACTAGG | 55 | 30 |
| LS68 | *A. fulvescens* | May et al., 1997 | (GATA)13 | U72739 | 128-160 | F: TTATTGCATGGTGTAGCTAAAC  R: AGCCCAACACAGACAATATC | 57 | 45 |
| LS39 | *A. fulvescens* | May et al., 1997 | (GTT)10 | U72734 | 120-123 | F: *TTCTGAAGTTCACACATTG  R: ATGGAGCATTATTGGAAGG | TD 67-57  (10 cycles)  57 (25 cycles) | 30 |
| Aox23 | *A. oxyrhinchus* | King et al., 2001 | (ATT)2(ACT)10  (AAT)5 | AF067811 | 91-133 | F: CAGTGTGCTAGCTTCTCAATA  R: GTTAGCTTAACCATGAATTGTG | 53 | 45 |
| Aox45 | *A. oxyrhinchus* | King et al., 2001 | (AAT)20 | AF067813 | 109-145 | F: TTGTCCAATAGTTTCCAACGC  R: TGTGCTCCTGCTTTTACTGTC | 53 | 45 |
| AoxD161 | *A. oxyrhinchus* | Henderson-Arzapalo et al, 2002 | (CTAT) 15 | AY093639 | 118-148 | F:GTTTGAAATGATTGAGAAAATGC  R:TGAGACAGACACTCTAGTTAAACAGC | 58 | 45 |
| Aox27 | *A. oxyrhinchus* | King et al.,2001 | (ATTT)5(ATT) (ATTT)3 | AF067812 | 134 | F: *AATAACAATAACGGCAGAACCT  R: TGTGTTGCTCAAGACAGTATGA | 60 | 45 |
| AoxD234 | *A. oxyrhinchus* | Henderson-Arzapalo et al, 2002 | (TAGA)17 | AY093645 | 195-322 | F:*AACTGGCTTTGTGATTGATCC  R:TGAAGCAAAGGGTATTATTTGAG | 52 | 30 |
| Spl100 | *S. plathorhynchus* | McQuown et al. 2000 | (TCTR)20 | AF276169 | 226 | F:CCATGCCTGTTCATAATTGTTC  R: TTTTCACCATTGGGTGTTCA | 57 | 45 |
| Spl104 | *S. plathorhynchus* | McQuown et al. 2000 | (TCTR)12 | AF276173 | 270 | F: TTATATGGGTGGGGTGGATG  R: TCCTCTTTGGCATTTGTTCC | 57 | 45 |
| Spl120 | *S. plathorhynchus* | McQuown et al. 2000 | (TATC)15 | AF276189 | 263-303 | F: ATTCCATGAGCAACACCACA  R: TGATGGTCTGATGAGATCGG | 55 | 45 |
| Spl106 | *S. plathorhynchus* | McQuown et al. 2000 | (CTAT)12 | AF276175 | 224 | F: CACGTGGATGCGAGAAATAC  R: GGGGAGAAAACTGGGGTAAA | 57 | 45 |
| Spl163 | *S. plathorhynchus* | McQuown et al. 2000 | (GATA)17 | AF276205 | 208 | F: CACTGATTCGCTACAACCGT  R:AGAAGGACTTGCAGTCCGAA | 57 | 45 |
| Spl101 | *S. plathorhynchus* | McQuown et al. 2000 | (TCTA)7 | AF276170 | 272 | F: CCCTCCACTGGAAATTTGAC  R: GCAATCAACAAGGTCTCTTTCA | 57 | 45 |
| AnacE4 | *A. naccari* | Forlani et al. 2007 | (CA)20 | EF576950 | 326-354 | F:* TCAGCTACAGGGTTCTGGG  R: GTTGTTACTCATTGGAACTC | 55 | 45 |
| AnacG8 | *A. naccari* | Forlani et al. 2007 | (CA)13 | EF576951 | 130-140 | F: GGTAGCAATAAGTGGAGTG  R: CTAGTTTCCCTTGATCAAGG | 52 | 30 |
| AnacC11 | *A. naccari* | Forlani et al. 2007 | (TCTA)12 | EF576948 | 167-193 | F: *AAATTTCCATTGGGGTGT  R: CTTCGTTTTGAGAACCCG | 50 | 45 |
| AnacB11 | *A. naccari* | Forlani et al. 2007 | (CA)9AA(CA)10 | EF576947 | 132-162 | F: GCAGCAGAATTCAGAACATG  R: TGATGGAACACAAGACAGTG | 55 | 30 |
| An20 | *A. naccari* | Zane et al., 2002 | (ATCT)10(TG)5 | AY144618 | 163-213 | F:AATAACAATCATTACATGAGGCT  R: TGGTCAGTTGTTTTTTTATTGAT | TD 58-54.5  (8 cycles)  54 (27 cycles) | 30 |
| As004 | *A. sinensis* | Zhu et al, 2005 | (CAAA)n | AY921010 | 102-200 | F: ACGAGTCCAAGTCCAGGTTG  R: AGGTCGAACCGCTAAAGGAC | 58 | 45 |
| As002 | *A. sinensis* | Zhu et al, 2005 | (GACA)n | AY921009 | 106-128 | F: CGGACAGAATTGGAGAACAC  R: TAACGCTGCCATTGCAGATA | 58 | 45 |
| AciG198 | *A.transmontantus* | Börk et al. 2007 | (AAAT)n | EF639163 | 160-200 | F: ACCATCCCCTCCCATATCTC  R: TGGTTCTTAGCGGAGGAAGA | 56 | 45 |
| AciG56 | *A.transmontantus* | Börk et al. 2007 | (TAGA)n | EF639135 | 280-320 | F: TGTTGCACTGTGTGCTGTGT  R: ACCCAAATCCTGCAGACAAG | 56 | 45 |

* Fluorescent labelled primers

Table ST2. Amplifications results for 25 tested microsatellite loci.

|  | Amplification results | | | | | | | | | | |  |
| --- | --- | --- | --- | --- | --- | --- | --- | --- | --- | --- | --- | --- |
| **Locus** | *H. huso* | *A. gueldenstaedtii* | | | | | *A. stellatus* | | *A. ruthenus* | | |  |
| LS19 | G/P | G/P | | | | | G/P | | G/P | | |  |
| LS34 | G/P | G/P | | | | | G/P | | G/P | | |  |
| LS 54 | G/P | G/P | | | | | G/P | | G/P | | |  |
| LS68 | NG | NG | | | | | NG | | NG | | |  |
| LS39 | G/P | G/P | | | | | G/P | | G/P | | |  |
| Aox23 | (-) | S | | | | | S | | (-) | | |  |
| Aox45 | G | NG | | | | | G | | G | | |  |
| AoxD161 | G | NG | | | | | G | | G | | |  |
| Aox27 | G/P | G/P | | | | | G/P | | G/P | | |  |
| AoxD234 | G/P | G/P | | | | | G/P | | G/P | | |  |
| Spl100 | G | G/P | | | | | G | | (-) | | |  |
| Spl104 | NG | G | | | | | G | | NG | | |  |
| Spl120 | NG | NG | | | | | NG | | NG | | |  |
| Spl106 | G/M | G/M | | | | | G/M | | G/M | | |  |
| Spl163 | NG | NG | | | | | NG | | NG | | |  |
| Spl101 | (-) | S | | | | | (-) | | (-) | | |  |
| AnacE4 | G/P | G/P | | | | | G/P | | G/P | | |  |
| AnacG8 | NG | NG | | | | | NG | | NG | | |  |
| AnacC11 | G/P | G/P | | | | | G/P | | G/P | | |  |
| AnacB11 | NG | NG | | | | | NG | | NG | | |  |
| An20 | NG | NG | | | | | NG | | NG | | |  |
| As004 | NG | NG | | | | | G | | G | | |  |
| As002 | G/M | G/M | | | | | G/M | | G/M | | |  |
| AciG198 | G/M | G/M | | | | | G/M | | G/M | | |  |
| AciG56 | S | NG | | | | | NG | | S | | |  |
| G = Good - clear and sharp bands  M = Monomorphic | | | |  | |  | |  | |  |  | |
| S = Stutter bands | | |  | |  |  | |  | |  |  | |
| P = Polymorphic | | | | | | | | | | | | |
| NG = Not Good multiple amplifications or individual alleles could not be distinguished correctly (difficult reading) | | | |  | |  | |  | |  |  | |
| (-) = no amplification | | | |  | |  | |  | |  |  | |

Table ST3. Results of the Bayesian assignment test done with STRUCTURE. High probabilities are listed in bold and the second probabilities are listed in bold italic.

| Individual N° | A. gueldenstaedtii | A. ruthenus | A. stellatus | H. huso |
| --- | --- | --- | --- | --- |
| Ast_1886_1 | 0.002 | 0.002 | 0.991 | 0.005 |
| Ast_4140_1 | 0.003 | 0.003 | 0.992 | 0.003 |
| Ast_8038_1 | 0.002 | 0.002 | 0.993 | 0.002 |
| Ast_7188_1 | 0.003 | 0.003 | 0.992 | 0.002 |
| Ast_9296_1 | 0.003 | 0.004 | 0.991 | 0.002 |
| Ast_1818_1 | 0.002 | 0.003 | 0.991 | 0.003 |
| Ast_4150_1 | 0.006 | 0.002 | 0.990 | 0.002 |
| Ast_0599_1 | 0.004 | 0.002 | 0.990 | 0.004 |
| Ast_6874_1 | 0.003 | 0.002 | 0.993 | 0.002 |
| Ast_6000_1 | 0.003 | 0.002 | 0.992 | 0.003 |
| Ast_9741_1 | 0.002 | 0.004 | 0.990 | 0.004 |
| Ast_274_1 | 0.002 | 0.002 | 0.993 | 0.003 |
| Ast_261_1 | 0.002 | 0.002 | 0.992 | 0.003 |
| Ast_262_1 | 0.009 | 0.002 | 0.987 | 0.002 |
| Ast_263_1 | 0.002 | 0.002 | 0.992 | 0.004 |
| Ast_264_1 | 0.002 | 0.002 | 0.994 | 0.002 |
| Ast_265_1 | 0.002 | 0.002 | 0.993 | 0.002 |
| Ast_266_1 | 0.004 | 0.004 | 0.990 | 0.002 |
| Ast_267_1 | 0.003 | 0.002 | 0.992 | 0.002 |
| Ast_268_1 | 0.003 | 0.002 | 0.993 | 0.002 |
| Ast_269_1 | 0.003 | 0.002 | 0.993 | 0.002 |
| Hh_7_4_2_1 | 0.003 | 0.002 | 0.002 | 0.993 |
| Hh_7_4_3_1 | 0.003 | 0.003 | 0.003 | 0.990 |
| Hh_7_11_13_ | 0.003 | 0.002 | 0.003 | 0.992 |
| Hh_7_11_3_1 | 0.003 | 0.002 | 0.002 | 0.993 |
| Hh_7_11_19_ | 0.003 | 0.002 | 0.002 | 0.993 |
| Hh_7_11_18_ | 0.004 | 0.002 | 0.002 | 0.992 |
| Hh_7_11_9_1 | 0.003 | 0.003 | 0.003 | 0.991 |
| Hh_7_11_17_ | 0.004 | 0.004 | 0.003 | 0.989 |
| Hh_7_11_12_ | 0.010 | 0.003 | 0.002 | 0.985 |
| Hh_7_11_21_ | 0.003 | 0.003 | 0.003 | 0.991 |
| Hh_7_11_15_ | 0.003 | 0.002 | 0.003 | 0.992 |
| Hh_247_1 | 0.039 | 0.006 | 0.015 | 0.940 |
| Hh_275_1 | 0.003 | 0.002 | 0.003 | 0.992 |
| Hh_276_1 | 0.008 | 0.004 | 0.005 | 0.987 |
| Hh_277_1 | 0.005 | 0.004 | 0.003 | 0.995 |
| Hh_278_1 | 0.011 | 0.002 | 0.009 | 0.980 |
| Hh_279_1 | 0.004 | 0.002 | 0.003 | 0.991 |
| Hh_280_1 | 0.011 | 0.000 | 0.000 | 0.989 |
| Hh_281_1 | 0.003 | 0.002 | 0.002 | 0.993 |
| Hh_282_1 | 0.004 | 0.002 | 0.002 | 0.991 |
| Hh_283_1 | 0.010 | 0.000 | 0.000 | 0.990 |
| Agu_8_19_3a | 0.991 | 0.004 | 0.003 | 0.003 |
| Agu_8_19_3b | 0.992 | 0.003 | 0.002 | 0.003 |
| Agu_8_18_9a | 0.996 | 0.005 | 0.004 | 0.004 |
| Agu_8_18_9b | 0.990 | 0.004 | 0.003 | 0.003 |
| Agu_8_17_18 | 0.991 | 0.003 | 0.003 | 0.003 |
| Agu_8_17_18 | 0.991 | 0.003 | 0.003 | 0.003 |
| Agu_8_17_18 | 0.991 | 0.003 | 0.003 | 0.003 |
| Agu_8_19_13 | 0.987 | 0.006 | 0.003 | 0.004 |
| Agu_8_19_13 | 0.972 | 0.022 | 0.003 | 0.003 |
| Agu_8_19_13 | 0.991 | 0.004 | 0.002 | 0.003 |
| Agu_8_18_19 | 0.988 | 0.007 | 0.003 | 0.003 |
| Agu_8_18_19 | 0.983 | 0.012 | 0.002 | 0.003 |
| Agu_8_18_19 | 0.990 | 0.004 | 0.002 | 0.003 |
| Agu_8_17_19 | 0.986 | 0.006 | 0.003 | 0.005 |
| Agu_8_17_19 | 0.990 | 0.003 | 0.002 | 0.004 |
| Agu_8_17_19 | 0.986 | 0.005 | 0.003 | 0.005 |
| Agu_8_17_13 | 0.991 | 0.003 | 0.003 | 0.003 |
| Agu_8_17_13 | 0.990 | 0.003 | 0.002 | 0.005 |
| Agu_8_17_13 | 0.990 | 0.003 | 0.003 | 0.005 |
| Agu_284a_1 | 0.992 | 0.003 | 0.002 | 0.003 |
| Agu_284b_1 | 0.992 | 0.003 | 0.003 | 0.003 |
| Agu_284c_1 | 0.993 | 0.003 | 0.002 | 0.003 |
| Agu_285a_1 | 0.989 | 0.003 | 0.004 | 0.004 |
| Agu_285b_1 | 0.984 | 0.004 | 0.006 | 0.006 |
| Agu_286a_1 | 0.989 | 0.003 | 0.003 | 0.004 |
| Agu_286b_1 | 0.989 | 0.003 | 0.004 | 0.004 |
| Agu_286c_1 | 0.990 | 0.003 | 0.004 | 0.003 |
| Agu_248_1 | 0.211 | 0.003 | 0.003 | 0.783 |
| Agu_259_1 | 0.643 | 0.004 | 0.344 | 0.009 |
| Agu_270_1 | 0.986 | 0.003 | 0.003 | 0.008 |
| Agu_271a_1 | 0.993 | 0.002 | 0.002 | 0.003 |
| Agu_271b_1 | 0.993 | 0.002 | 0.002 | 0.003 |
| Agu_271c_1 | 0.993 | 0.002 | 0.002 | 0.003 |
| Agu_272a_1 | 0.989 | 0.005 | 0.003 | 0.003 |
| Agu_272b_1 | 0.989 | 0.005 | 0.003 | 0.003 |
| Agu_272c_1 | 0.989 | 0.005 | 0.003 | 0.003 |
| Agu_273a_1 | 0.991 | 0.003 | 0.002 | 0.004 |
| Agu_273b_1 | 0.992 | 0.003 | 0.002 | 0.003 |
| Agu_273c_1 | 0.992 | 0.003 | 0.002 | 0.003 |
| Aru_7_10_9_ | 0.006 | 0.994 | 0.003 | 0.002 |
| Aru_7_10_8_ | 0.003 | 0.993 | 0.002 | 0.002 |
| Aru_7_10_7_ | 0.003 | 0.992 | 0.002 | 0.003 |
| Aru_8_1_11_ | 0.004 | 0.992 | 0.002 | 0.002 |
| Aru_8_1_12_ | 0.003 | 0.992 | 0.003 | 0.002 |
| Aru_8_1_7_1 | 0.002 | 0.994 | 0.002 | 0.002 |
| Aru_249_1 | 0.002 | 0.994 | 0.002 | 0.002 |
| Aru_250_1 | 0.004 | 0.992 | 0.002 | 0.002 |
| Aru_251_1 | 0.003 | 0.993 | 0.002 | 0.002 |
| Aru_252_1 | 0.003 | 0.993 | 0.002 | 0.003 |
| Aru_253_1 | 0.003 | 0.993 | 0.002 | 0.002 |
| Aru_254_1 | 0.005 | 0.991 | 0.002 | 0.002 |
| Aru_255_1 | 0.004 | 0.991 | 0.003 | 0.003 |
| Aru_256_1 | 0.003 | 0.989 | 0.003 | 0.005 |
| Aru_257_1 | 0.00 4 | 0.991 | 0.002 | 0.003 |
| Aru_258_1 | 0.003 | 0.993 | 0.002 | 0.002 |
| 16_15 | 0.066 | 0.004 | 0.009 | 0.923 |
| B2 | 0.007 | 0.002 | 0.003 | 0.993 |
| 3_6_2 | 0.004 | 0.002 | 0.980 | 0.020 |
| 3_6_3 | 0.004 | 0.005 | 0.920 | 0.075 |
| 3_6_4 | 0.005 | 0.010 | 0.472 | 0.523 |
| 3_5_1 | 0.003 | 0.002 | 0.965 | 0.035 |
| 3_6_11 | 0.014 | 0.003 | 0.792 | 0.194 |
| 3_7_8 | 0.003 | 0.002 | 0.991 | 0.009 |
| 2_6_10 | 0.014 | 0.003 | 0.842 | 0.146 |
| 2_6_27 | 0.021 | 0.002 | 0.974 | 0.006 |

**Table ST4.** Posterior probability values for *A. stellatus, H. huso* and their hybrids.

|  | Pure H. huso | Pure A. stellatus | F1 | F2 | Bc1 | Bc2 |
| --- | --- | --- | --- | --- | --- | --- |
| Ast_1886 | 0.000 | 0.969 | 0.000 | 0.000 | 0.000 | 0.031 |
| Ast_4140 | 0.000 | 0.994 | 0.000 | 0.000 | 0.000 | 0.006 |
| Ast_8038 | 0.000 | 0.996 | 0.000 | 0.000 | 0.000 | 0.004 |
| Ast_7188 | 0.000 | 0.995 | 0.000 | 0.000 | 0.000 | 0.005 |
| Ast_9296 | 0.000 | 0.997 | 0.000 | 0.000 | 0.000 | 0.003 |
| Ast_1818 | 0.000 | 0.987 | 0.000 | 0.000 | 0.000 | 0.013 |
| Ast_4150 | 0.000 | 0.996 | 0.000 | 0.000 | 0.000 | 0.004 |
| Ast_0599 | 0.000 | 0.972 | 0.000 | 0.000 | 0.000 | 0.028 |
| Ast_6874 | 0.000 | 0.996 | 0.000 | 0.000 | 0.000 | 0.004 |
| Ast_6000 | 0.000 | 0.990 | 0.000 | 0.000 | 0.000 | 0.009 |
| Ast_9741 | 0.000 | 0.971 | 0.000 | 0.000 | 0.000 | 0.029 |
| Ast_274 | 0.000 | 0.993 | 0.000 | 0.000 | 0.000 | 0.007 |
| Ast_261 | 0.000 | 0.987 | 0.000 | 0.000 | 0.000 | 0.013 |
| Ast_262 | 0.000 | 0.997 | 0.000 | 0.000 | 0.000 | 0.003 |
| Ast_263 | 0.000 | 0.983 | 0.000 | 0.000 | 0.000 | 0.017 |
| Ast_264 | 0.000 | 0.997 | 0.000 | 0.000 | 0.000 | 0.003 |
| Ast_265 | 0.000 | 0.995 | 0.000 | 0.000 | 0.000 | 0.005 |
| Ast_266 | 0.000 | 0.995 | 0.000 | 0.000 | 0.000 | 0.005 |
| Ast_267 | 0.000 | 0.995 | 0.000 | 0.000 | 0.000 | 0.005 |
| Ast_268 | 0.000 | 0.998 | 0.000 | 0.000 | 0.000 | 0.002 |
| Ast_269 | 0.000 | 0.996 | 0.000 | 0.000 | 0.000 | 0.004 |
| Hh_7_4_2 | 1.000 | 0.000 | 0.000 | 0.000 | 0.000 | 0.000 |
| Hh_7_4_3 | 0.999 | 0.000 | 0.000 | 0.000 | 0.000 | 0.000 |
| Hh_7_11_13 | 1.000 | 0.000 | 0.000 | 0.000 | 0.000 | 0.000 |
| Hh_7_11_3 | 1.000 | 0.000 | 0.000 | 0.000 | 0.000 | 0.000 |
| Hh_7_11_19 | 1.000 | 0.000 | 0.000 | 0.000 | 0.000 | 0.000 |
| Hh_7_11_18 | 1.000 | 0.000 | 0.000 | 0.000 | 0.000 | 0.000 |
| Hh_7_11_9 | 1.000 | 0.000 | 0.000 | 0.000 | 0.000 | 0.000 |
| Hh_7_11_17 | 1.000 | 0.000 | 0.000 | 0.000 | 0.000 | 0.000 |
| Hh_7_11_12 | 1.000 | 0.000 | 0.000 | 0.000 | 0.000 | 0.000 |
| Hh_7_11_21 | 1.000 | 0.000 | 0.000 | 0.000 | 0.000 | 0.000 |
| Hh_7_11_15 | 0.977 | 0.000 | 0.000 | 0.006 | 0.017 | 0.000 |
| Hh_247 | 0.987 | 0.000 | 0.000 | 0.006 | 0.007 | 0.000 |
| Hh_275 | 1.000 | 0.000 | 0.000 | 0.000 | 0.000 | 0.000 |
| Hh_276 | 0.999 | 0.000 | 0.000 | 0.000 | 0.000 | 0.000 |
| Hh_277 | 1.000 | 0.000 | 0.000 | 0.000 | 0.000 | 0.000 |
| Hh_278 | 1.000 | 0.000 | 0.000 | 0.000 | 0.000 | 0.000 |
| Hh_279 | 1.000 | 0.000 | 0.000 | 0.000 | 0.000 | 0.000 |
| Hh_280 | 1.000 | 0.000 | 0.000 | 0.000 | 0.000 | 0.000 |
| Hh_281 | 1.000 | 0.000 | 0.000 | 0.000 | 0.000 | 0.000 |
| Hh_282 | 1.000 | 0.000 | 0.000 | 0.000 | 0.000 | 0.000 |
| Hh_283 | 1.000 | 0.000 | 0.000 | 0.000 | 0.000 | 0.000 |
| 3_6_2 | 0 | 0.00008 | 0.6806 | 0.03201 | 0.00092 | 0.28638 |
| 3_6_3 | 0 | 0.00001 | 0.1172 | 0.17105 | 0.00007 | 0.71163 |
| 3_6_4 | 0 | 0 | 0.9586 | 0.0213067 | 0.01073 | 0.009347 |
| 3_5_1 | 0 | 0.00003 | 0.8193 | 0.0145667 | 0.00116 | 0.164987 |
| 3_6_11 | 0 | 0.00001 | 0.2034 | 0.11948 | 0.00011 | 0.67697 |
| 3_7_8 | 0 | 0.00233 | 0.0061 | 0.29325 | 0.00008 | 0.69821 |
| 2_6_10 | 0 | 0.001385 | 0.3076 | 0.193425 | 0.00078 | 0.496845 |
| 2_6_27 | 0 | 0.015626667 | 0.244 | 0.08054 | 0.00032 | 0.65951 |

Table ST5. Posterior probability values for A. gueldenstaedtii, H. huso and their hybrids.

|  | Pure Agu | Pure Hh | F1 | F2 | Bc1 | Bc2 |
| --- | --- | --- | --- | --- | --- | --- |
| Hh_7_4_2 | 0.000 | 0.995 | 0.000 | 0.000 | 0.000 | 0.005 |
| Hh_7_4_3 | 0.000 | 0.990 | 0.000 | 0.000 | 0.000 | 0.010 |
| Hh_7_11_13 | 0.000 | 0.993 | 0.000 | 0.000 | 0.000 | 0.007 |
| Hh_7_11_3 | 0.000 | 0.996 | 0.000 | 0.000 | 0.000 | 0.004 |
| Hh_7_11_19 | 0.000 | 0.995 | 0.000 | 0.000 | 0.000 | 0.005 |
| Hh_7_11_18 | 0.000 | 0.977 | 0.000 | 0.000 | 0.000 | 0.023 |
| Hh_7_11_9 | 0.000 | 0.993 | 0.000 | 0.000 | 0.000 | 0.007 |
| Hh_7_11_17 | 0.000 | 0.972 | 0.000 | 0.000 | 0.000 | 0.027 |
| Hh_7_11_12 | 0.000 | 0.904 | 0.000 | 0.004 | 0.000 | 0.092 |
| Hh_7_11_21 | 0.000 | 0.991 | 0.000 | 0.000 | 0.000 | 0.009 |
| Hh_7_11_15 | 0.000 | 0.992 | 0.000 | 0.000 | 0.000 | 0.008 |
| Hh_247 | 0.000 | 0.878 | 0.000 | 0.004 | 0.000 | 0.119 |
| Hh_275 | 0.000 | 0.992 | 0.000 | 0.000 | 0.000 | 0.008 |
| Hh_276 | 0.000 | 0.880 | 0.000 | 0.000 | 0.000 | 0.120 |
| Hh_277 | 0.000 | 0.944 | 0.000 | 0.000 | 0.000 | 0.056 |
| Hh_278 | 0.000 | 0.855 | 0.000 | 0.001 | 0.000 | 0.144 |
| Hh_279 | 0.000 | 0.991 | 0.000 | 0.000 | 0.000 | 0.009 |
| Hh_280 | 0.000 | 0.904 | 0.000 | 0.007 | 0.000 | 0.088 |
| Hh_281 | 0.000 | 0.990 | 0.000 | 0.000 | 0.000 | 0.010 |
| Hh_282 | 0.000 | 0.983 | 0.000 | 0.000 | 0.000 | 0.017 |
| Hh_283 | 0.000 | 0.922 | 0.000 | 0.002 | 0.000 | 0.076 |
| Agu_8_19_3a | 1.000 | 0.000 | 0.000 | 0.000 | 0.000 | 0.000 |
| Agu_8_19_3b | 0.999 | 0.000 | 0.000 | 0.000 | 0.001 | 0.000 |
| Agu_8_18_9a | 1.000 | 0.000 | 0.000 | 0.000 | 0.000 | 0.000 |
| Agu_8_18_9b | 1.000 | 0.000 | 0.000 | 0.000 | 0.000 | 0.000 |
| Agu_8_17_18a | 0.999 | 0.000 | 0.000 | 0.000 | 0.001 | 0.000 |
| Agu_8_17_18b | 0.999 | 0.000 | 0.000 | 0.000 | 0.001 | 0.000 |
| Agu_8_17_18c | 0.999 | 0.000 | 0.000 | 0.000 | 0.001 | 0.000 |
| Agu_8_19_13a | 0.999 | 0.000 | 0.000 | 0.000 | 0.001 | 0.000 |
| Agu_8_19_13b | 1.000 | 0.000 | 0.000 | 0.000 | 0.000 | 0.000 |
| Agu_8_19_13c | 1.000 | 0.000 | 0.000 | 0.000 | 0.000 | 0.000 |
| Agu_8_18_19a | 0.999 | 0.000 | 0.000 | 0.000 | 0.001 | 0.000 |
| Agu_8_18_19b | 1.000 | 0.000 | 0.000 | 0.000 | 0.000 | 0.000 |
| Agu_8_18_19c | 0.999 | 0.000 | 0.000 | 0.000 | 0.001 | 0.000 |
| Agu_8_17_19a | 0.999 | 0.000 | 0.000 | 0.000 | 0.001 | 0.000 |
| Agu_8_17_19b | 0.999 | 0.000 | 0.000 | 0.000 | 0.001 | 0.000 |
| Agu_8_17_19c | 0.998 | 0.000 | 0.000 | 0.000 | 0.001 | 0.000 |
| Agu_8_17_13a | 0.999 | 0.000 | 0.000 | 0.000 | 0.001 | 0.000 |
| Agu_8_17_13b | 0.999 | 0.000 | 0.000 | 0.000 | 0.001 | 0.000 |
| Agu_8_17_13c | 0.999 | 0.000 | 0.000 | 0.000 | 0.001 | 0.000 |
| Agu_284a | 1.000 | 0.000 | 0.000 | 0.000 | 0.000 | 0.000 |
| Agu_284b | 1.000 | 0.000 | 0.000 | 0.000 | 0.000 | 0.000 |
| Agu_284c | 1.000 | 0.000 | 0.000 | 0.000 | 0.000 | 0.000 |
| Agu_285a | 0.999 | 0.000 | 0.000 | 0.000 | 0.001 | 0.000 |
| Agu_285b | 0.999 | 0.000 | 0.000 | 0.000 | 0.001 | 0.000 |
| Agu_286a | 0.999 | 0.000 | 0.000 | 0.000 | 0.001 | 0.000 |
| Agu_286b | 0.999 | 0.000 | 0.000 | 0.000 | 0.001 | 0.000 |
| Agu_286c | 0.999 | 0.000 | 0.000 | 0.000 | 0.001 | 0.000 |
| Agu_248 | 0.017 | 0.155 | 0.013 | 0.168 | 0.014 | 0.634 |
| Agu_259 | 0.803 | 0.000 | 0.001 | 0.067 | 0.128 | 0.001 |
| Agu_270 | 0.999 | 0.000 | 0.000 | 0.000 | 0.001 | 0.000 |
| Agu_271a | 1.000 | 0.000 | 0.000 | 0.000 | 0.000 | 0.000 |
| Agu_271b | 1.000 | 0.000 | 0.000 | 0.000 | 0.000 | 0.000 |
| Agu_271c | 1.000 | 0.000 | 0.000 | 0.000 | 0.000 | 0.000 |
| Agu_272a | 0.999 | 0.000 | 0.000 | 0.000 | 0.001 | 0.000 |
| Agu_272b | 0.999 | 0.000 | 0.000 | 0.000 | 0.001 | 0.000 |
| Agu_272c | 0.999 | 0.000 | 0.000 | 0.000 | 0.001 | 0.000 |
| Agu_273a | 1.000 | 0.000 | 0.000 | 0.000 | 0.000 | 0.000 |
| Agu_273b | 1.000 | 0.000 | 0.000 | 0.000 | 0.000 | 0.000 |
| Agu_273c | 1.000 | 0.000 | 0.000 | 0.000 | 0.000 | 0.000 |
| 16_15 | 0.000 | 0.462 | 0.000 | 0.014 | 0.000 | 0.523 |

Table ST6. Posterior probability values for *A. stellatus, A. gueldenstaedtii* and
their hybrids.

|  | Pure Ast | Pure Agu | F1 | F2 | Bc1 | Bc2 |
| --- | --- | --- | --- | --- | --- | --- |
| Ast_1886 | 1.000 | 0.000 | 0.000 | 0.000 | 0.000 | 0.000 |
| Ast_4140 | 1.000 | 0.000 | 0.000 | 0.000 | 0.000 | 0.000 |
| Ast_8038 | 1.000 | 0.000 | 0.000 | 0.000 | 0.000 | 0.000 |
| Ast_7188 | 1.000 | 0.000 | 0.000 | 0.000 | 0.000 | 0.000 |
| Ast_9296 | 1.000 | 0.000 | 0.000 | 0.000 | 0.000 | 0.000 |
| Ast_1818 | 1.000 | 0.000 | 0.000 | 0.000 | 0.000 | 0.000 |
| Ast_4150 | 0.998 | 0.000 | 0.000 | 0.001 | 0.001 | 0.000 |
| Ast_0599 | 0.999 | 0.000 | 0.000 | 0.001 | 0.001 | 0.000 |
| Ast_6874 | 1.000 | 0.000 | 0.000 | 0.000 | 0.000 | 0.000 |
| Ast_6000 | 1.000 | 0.000 | 0.000 | 0.000 | 0.000 | 0.000 |
| Ast_9741 | 1.000 | 0.000 | 0.000 | 0.000 | 0.000 | 0.000 |
| Ast_274 | 1.000 | 0.000 | 0.000 | 0.000 | 0.000 | 0.000 |
| Ast_261 | 1.000 | 0.000 | 0.000 | 0.000 | 0.000 | 0.000 |
| Ast_262 | 0.992 | 0.000 | 0.000 | 0.006 | 0.002 | 0.000 |
| Ast_263 | 1.000 | 0.000 | 0.000 | 0.000 | 0.000 | 0.000 |
| Ast_264 | 1.000 | 0.000 | 0.000 | 0.000 | 0.000 | 0.000 |
| Ast_265 | 1.000 | 0.000 | 0.000 | 0.000 | 0.000 | 0.000 |
| Ast_266 | 0.999 | 0.000 | 0.000 | 0.001 | 0.001 | 0.000 |
| Ast_267 | 1.000 | 0.000 | 0.000 | 0.000 | 0.000 | 0.000 |
| Ast_268 | 1.000 | 0.000 | 0.000 | 0.000 | 0.000 | 0.000 |
| Ast_269 | 1.000 | 0.000 | 0.000 | 0.000 | 0.000 | 0.000 |
| Agu_8_19_3a | 0.000 | 1.000 | 0.000 | 0.000 | 0.000 | 0.000 |
| Agu_8_19_3b | 0.000 | 1.000 | 0.000 | 0.000 | 0.000 | 0.000 |
| Agu_8_18_9a | 0.000 | 1.000 | 0.000 | 0.000 | 0.000 | 0.000 |
| Agu_8_18_9b | 0.000 | 1.000 | 0.000 | 0.000 | 0.000 | 0.000 |
| Agu_8_17_18a | 0.000 | 1.000 | 0.000 | 0.000 | 0.000 | 0.000 |
| Agu_8_17_18b | 0.000 | 1.000 | 0.000 | 0.000 | 0.000 | 0.000 |
| Agu_8_17_18c | 0.000 | 1.000 | 0.000 | 0.000 | 0.000 | 0.000 |
| Agu_8_19_13a | 0.000 | 1.000 | 0.000 | 0.000 | 0.000 | 0.000 |
| Agu_8_19_13b | 0.000 | 1.000 | 0.000 | 0.000 | 0.000 | 0.000 |
| Agu_8_19_13c | 0.000 | 1.000 | 0.000 | 0.000 | 0.000 | 0.000 |
| Agu_8_18_19a | 0.000 | 1.000 | 0.000 | 0.000 | 0.000 | 0.000 |
| Agu_8_18_19b | 0.000 | 1.000 | 0.000 | 0.000 | 0.000 | 0.000 |
| Agu_8_18_19c | 0.000 | 1.000 | 0.000 | 0.000 | 0.000 | 0.000 |
| Agu_8_17_19a | 0.000 | 1.000 | 0.000 | 0.000 | 0.000 | 0.000 |
| Agu_8_17_19b | 0.000 | 1.000 | 0.000 | 0.000 | 0.000 | 0.000 |
| Agu_8_17_19c | 0.000 | 1.000 | 0.000 | 0.000 | 0.000 | 0.000 |
| Agu_8_17_13a | 0.000 | 1.000 | 0.000 | 0.000 | 0.000 | 0.000 |
| Agu_8_17_13b | 0.000 | 1.000 | 0.000 | 0.000 | 0.000 | 0.000 |
| Agu_8_17_13c | 0.000 | 1.000 | 0.000 | 0.000 | 0.000 | 0.000 |
| Agu_284a | 0.000 | 1.000 | 0.000 | 0.000 | 0.000 | 0.000 |
| Agu_284b | 0.000 | 1.000 | 0.000 | 0.000 | 0.000 | 0.000 |
| Agu_284c | 0.000 | 1.000 | 0.000 | 0.000 | 0.000 | 0.000 |
| Agu_285a | 0.000 | 0.999 | 0.000 | 0.000 | 0.000 | 0.000 |
| Agu_285b | 0.000 | 0.998 | 0.000 | 0.001 | 0.000 | 0.001 |
| Agu_286a | 0.000 | 0.999 | 0.000 | 0.000 | 0.000 | 0.000 |
| Agu_286b | 0.000 | 0.999 | 0.000 | 0.001 | 0.000 | 0.000 |
| Agu_286c | 0.000 | 0.999 | 0.000 | 0.000 | 0.000 | 0.000 |
| Agu_248 | 0.000 | 0.984 | 0.000 | 0.015 | 0.000 | 0.001 |
| Agu_259 | 0.001 | 0.006 | 0.000 | 0.990 | 0.000 | 0.003 |
| Agu_270 | 0.000 | 1.000 | 0.000 | 0.000 | 0.000 | 0.000 |
| Agu_271a | 0.000 | 1.000 | 0.000 | 0.000 | 0.000 | 0.000 |
| Agu_271b | 0.000 | 1.000 | 0.000 | 0.000 | 0.000 | 0.000 |
| Agu_271c | 0.000 | 1.000 | 0.000 | 0.000 | 0.000 | 0.000 |
| Agu_272a | 0.000 | 1.000 | 0.000 | 0.000 | 0.000 | 0.000 |
| Agu_272b | 0.000 | 1.000 | 0.000 | 0.000 | 0.000 | 0.000 |
| Agu_272c | 0.000 | 1.000 | 0.000 | 0.000 | 0.000 | 0.000 |
| Agu_273a | 0.000 | 1.000 | 0.000 | 0.000 | 0.000 | 0.000 |
| Agu_273b | 0.000 | 1.000 | 0.000 | 0.000 | 0.000 | 0.000 |
| Agu_273c | 0.000 | 1.000 | 0.000 | 0.000 | 0.000 | 0.000 |
| 2_6_27a | 0.978 | 0.000 | 0.000 | 0.008 | 0.014 | 0.000 |
